# Circadian and Seasonal Pattern of Arrhythmic Events in Arrhythmogenic Cardiomyopathy Patients

**DOI:** 10.3390/ijerph20042872

**Published:** 2023-02-07

**Authors:** Silvia Castelletti, Michele Orini, Annina S. Vischer, William J. McKenna, Pier D. Lambiase, Antonios Pantazis, Lia Crotti

**Affiliations:** 1 Istituto Auxologico Italiano, IRCCS, Department of Cardiology, Piazzale Brescia 20, 20149 Milan, Italy; 2 Institute of Cardiovascular Science, University College London, London WC1E 6BT, UK; 3 Medical Outpatient Department, ESH Hypertension Centre of Excellence, University Hospital Basel, 4031 Basel, Switzerland; 4 Faculty of Medicine, University of Basel, 4056 Basel, Switzerland; 5 Department of Cardiology, University of A Coruña, 15001 A Coruña, Spain; 6 The Barts Heart Centre, Barts Health NHS Trust, London E1 1BB, UK; 7 National Heart and Lung Institute, Imperial College London, London SW7 2BX, UK; 8 Cardiovascular Research Centre, Royal Brompton and Harefield Hospitals, London SW3 6NP, UK; 9 Department of Medicine and Surgery, University of Milano-Bicocca, 20126 Milan, Italy

**Keywords:** arrhythmia, sudden death, circadian, daily, seasonal, arrhythmogenic cardiomyopathy, implantable cardioverter defibrillator, ventricular tachycardia

## Abstract

Arrhythmogenic right ventricular cardiomyopathy (ARVC) is an inherited cardiac disease associated with an increased risk of life-threatening arrhythmias. The aim of the present study was to evaluate the association of ventricular arrhythmias (VA) with circadian and seasonal variation in ARVC. One hundred two ARVC patients with an implantable cardioverter defibrillator (ICD) were enrolled in the study. Arrhythmic events included (a) any initial ventricular tachycardia (VT) or fibrillation (VF) prompting ICD implantation, (b) any VT or non-sustained VT (NSVT) recorded by the ICD, and (c) appropriate ICD shocks/therapy. Differences in the annual incidence of events across seasons (winter, spring, summer, autumn) and period of the day (night, morning, afternoon, evening) were assessed both for all cardiac events and major arrhythmic events. In total, 67 events prior to implantation and 263 ICD events were recorded. These included 135 major (58 ICD therapies, 57 self-terminating VT, 20 sustained VT) and 148 minor (NSVT) events. A significant increase in the frequency of events was observed in the afternoon versus in the nights and mornings (*p* = 0.016). The lowest number of events was registered in the summer, with a peak in the winter (*p* < 0.001). Results were also confirmed when excluding NSVT. Arrhythmic events in ARVC follow a seasonal variation and a circadian rhythm. They are more prevalent in the late afternoon, the most active period of the day, and in the winter, supporting the role of physical activity and inflammation as triggers of events.

## 1. Introduction

Arrhythmogenic right ventricular cardiomyopathy (ARVC) is an inherited cardiac condition that may result in life-threatening arrhythmia and sudden cardiac death (SCD), particularly in young adults, with an annual mortality estimated to be up to 25% in affected individuals < 30 years of age [1,2]. The triggers of these events are poorly understood, though there is a strong association between physical activity and development of arrhythmia [3]. A previous study reported a peak of occurrence of ventricular arrhythmia (VA) in ARVC patients in periods with higher humidity and temperatures [4]. These results have not been validated in ARVC and differ from the reported patterns of arrhythmic events in non-arrhythmogenic cardiomyopathy (non-ACM) populations, who exhibit peaks mainly in the winter time [5,6]. It is also well-documented that arrhythmia and SCD have a circadian pattern, with a peak in the morning in other cardiovascular diseases [7,8] and in the afternoon in hypertrophic cardiomyopathy (HCM) [9,10,11]. It is unknown whether the VAs in ACM patients follow a circadian pattern. 

We sought to determine whether the arrhythmic events in ACM patients with ARVC are associated with circadian and seasonal patterns.

## 2. Materials and Methods

### 2.1. Study Population and Clinical Assessment

The study population included 102 patients fulfilling the diagnostic criteria for ARVC (Table 1) who received an implantable cardioverter defibrillator (ICD). All patients were diagnosed according to the revised Task Force Criteria [1] and the last published criteria [12,13] at the former The Heart Hospital, at Westmoreland Street, University College London Hospitals (UCLH) NHS Foundation Trust, London, UK, through a previously detailed clinical assessment [14,15]. None of the patients were engaged in competitive sports at the time of the study.

Arrhythmic events included (a) any initial ventricular tachycardia (VT) or ventricular fibrillation (VF) prompting ICD implantation, (b) VT or non-sustained VT (NSVT) recorded by the ICD and (c) any appropriated ICD shocks/therapy.

Non-sustained ventricular tachycardia (NSVT) was defined as 3 or more consecutive ventricular beats with an RR interval of <600 ms (>120 bpm) and lasting <30 s, while lasting ≥ 30 s was defined as sustained ventricular tachycardia (VT). ICD appropriate interventions were defined as any ventricular arrhythmia treated by the device with anti-tachycardia pacing and/or shock. We defined NSVT as a minor event, all the others were defined as major events.

Left ventricular dysfunction was defined as ejection fraction (EF) ≤ 55% [16].

The intracardiac electrograms were reviewed by a single operator (S.C.): each of the stored electrograms had data designating the time of the day and the date of the arrhythmic event, and data were recorded over a period of 12 years, with a minimum of 12 months of follow-up to ensure a complete seasonal cycle for each patient.

For circadian analysis, the number of events were considered hour by hour and by phases of the day, defined according to the full rotation of the Earth with respect to the Sun and the commonly accepted work–leisure period of the day as night (00.00–6.00 am), morning (6.00–12.00 am), afternoon (12.00 am–6 pm) and evening (6–12 pm). Seasons were defined as: winter (January–March), spring (April–June), summer (July–September) and autumn (October–December).

All subjects gave their informed consent.

### 2.2. Statistical Analysis

Analyses were performed using the statistical software SPSS Statistics, version 21.0 (IBM Co, Armonk, NY, USA) and MATLAB R2019b (Mathwork). Continuous variables were expressed as mean and standard deviation or as median and interquartile range (IQR, 25th–75th percentile) whenever the distribution was skewed. Group comparisons in continuous variables were performed with either the Student’s *t*-test or with the Mann–Whitney U test, as appropriate. Categorical variables were presented as absolute (n) and relative frequencies (%) and compared among groups with either Fisher’s exact test or χ^2^ test, as appropriate. A 2-sided *p* value < 0.05 was considered statistically significant. The number of events occurring each year during a given season (winter, spring, summer, autumn) or period of the day (morning, afternoon, evening, night) was registered, and the annual incidence was computed. Differences in the annual incidence of events across seasons and period of the day were assessed using the Kruskal–Wallis test, while pair-wise comparisons were performed using the Wilcoxon sign-rank test for matched comparisons. Years for which fewer than 3 events were available were excluded.

## 3. Results

### 3.1. Study Population

The study population included 102 clinically confirmed ARVC patients (Table 1) with a transvenous ICD. Probands were overrepresented (75, 74%), and all but six were Caucasians (96, 94%). There were similar representations of both genders (55 males, 54%). The mean age at diagnosis was 40 ± 14 years, and the mean age at ICD implantation was 41 ± 15 years (Table 2). During the workup, a history of syncope was present in 35 (34%), and a family history of SCD < 35 years of age was present in 20 of the 75 probands (27%). Ten (10%) had an impaired left ventricular ejection fraction at the first evaluation. An ICD was implanted as primary and secondary prevention in 66 (65%) and 36 (35%) patients, respectively. In total, 67 events (47 VT/VF, 20 NSVT) prior to implantation and 263 ICD events (58 appropriate shocks/therapy, 148 NSVT, 57 self-terminating VT) were recorded and analysed. Eighteen patients (18%) had an appropriate ICD intervention, and, of these, seventeen (17%) had more than one appropriate ICD intervention, including one patient with four interventions and one patient with seven interventions. Of these patients, three had an electrical storm occur: one in winter, one in the afternoon and one during the night over two consecutive days. 

### 3.2. Circadian Pattern

**Figure 1:**
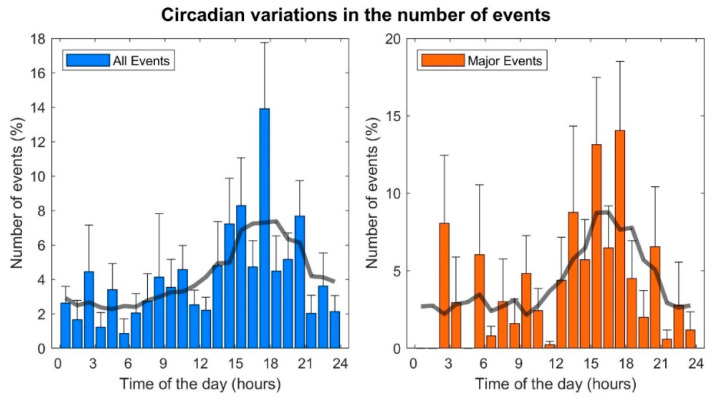
Distribution of events during the day. Bars: average hourly incidence of events computed across different years. Whiskers: standard error. Solid line: moving average.

**Figure 2:**
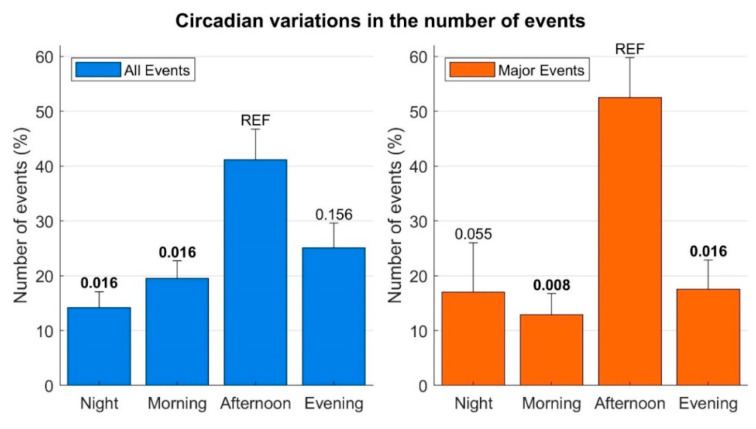
Distribution of the events by period of the day: night (00.00–6.00 am), morning (6.00–12.00 am), afternoon (12.00 am–6 pm) and evening (6–12 pm). Bars: number of events, averaged across years. Whiskers: standard error. *p*-values (Wilcoxon signed-rank test) assessing the difference between number of events registered during night, morning and evening with respect to the afternoon are shown. *p* < 0.05 is reported in bold. REF = reference.

The number of events was low during the nights and mornings, increased in the afternoon, peaked between 5–6 pm and then decreased during the evening (Figure 1). A similar pattern was observed when only including major events (Figure 1). The distribution of the number of events across the four main periods of the day (morning, afternoon, evening, night) was non-uniform (*p* = 0.0015). A significant increase in the frequency of events was observed in the afternoon compared to the nights and mornings (*p* = 0.016, Figure 2). The number of events registered during the evening was also lower than during the afternoon, but the difference was nonsignificant (*p* = 0.156). Similar results were found when only including major events (Figure 2).

### 3.3. Seasonal Variation

**Figure 3:**
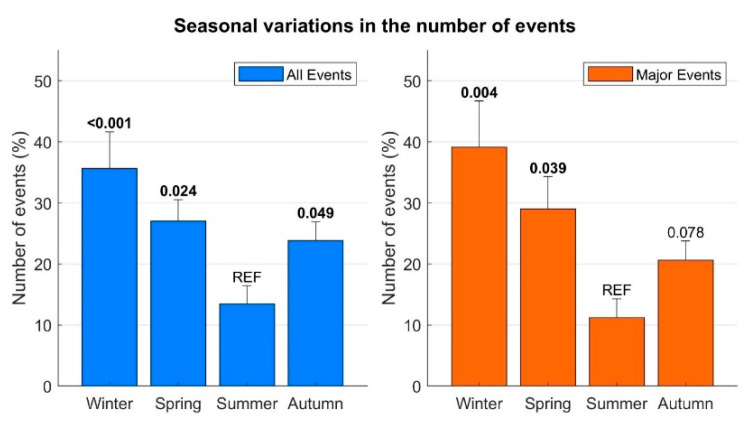
Seasonal distribution of the events. Bars: number of events, averaged across years. Whiskers: standard errors. *p*-values (Wilcoxon signed-rank test) assessing the difference between number of events registered during winter, spring and autumn with respect to those recorded during the summer (REF) are shown. *p* < 0.05 is reported in bold.

**Figure 4:**
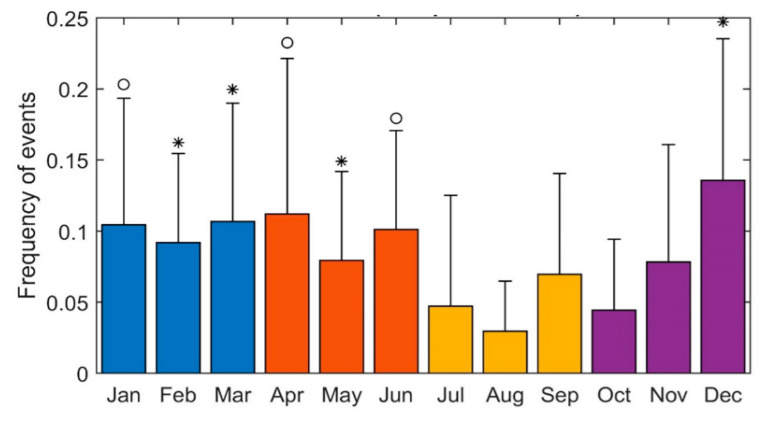
Distribution of event frequency for each month. The lowest event frequency was registered in August. Pairwise comparison shows that the event frequency registered in August was significantly lower than the event frequency in December, February, March and May (* indicates *p* < 0.05, ”◦” indicates 0.1 < *p* < 0.05, Wilcoxon ranksum test).

As shown in Figure 3, the frequency of events was inhomogeneous over the year’s four seasons (*p* = 0.0015). The lowest number of events was registered in the summer, with an increase in the autumn (*p* = 0.049), a peak in the winter (*p* < 0.001) and a decrease in the spring, with the average number of events still significantly higher than during the summer (*p* = 0.024). These results did not change even when considering a stricter definition of the seasons, according to the exact day of the solstices and equinoxes. This seasonal distribution was also confirmed when considering only the major arrhythmic events (excluding NSVT) (group differences *p* = 0.003, Figure 3). This was also confirmed by analysing the events for each month (Figure 4): the number of events was significantly lower in August and had a peak in December.

## 4. Discussion

### 4.1. Circadian and Seasonal Determinants of Biological Internal Clocks

Our universe exhibits important cyclical variations, with one of the most well-known being the influence of the moon on the Earth’s oceans and their tides. Human life cycles are also influenced by biological variations related to the time of day or night as well as the seasons.

This study is the largest so far published that evaluates circadian and seasonal variations of arrhythmic events in ARVC. ICDs store detailed information on the occurrence (time and day) of VA, providing a unique opportunity to accurately assess the precise timing of these arrhythmic events. 

There are two main findings of our study:

- Arrhythmic events in ARVC are significantly more frequent in the afternoon, around 6 pm;
- Arrhythmic events occur more frequently during the winter and less frequently in the summer.

The cardiovascular system’s circadian clock regulates the heart and vasculature to prevent deleterious variations in response to stimuli during the day/night.

Malfunction of the circadian and seasonal cycles may impair the cardiovascular system’s ability to respond appropriately to environmental stimuli. In particular, impairment of the circadian system may promote inflammation with increased inflammatory markers as well as altering hormonal balance and central nervous system modulation with increased sympathetic activity, calcium overload potentially promoting delayed afterdepolarisations and arrhythmia [6,7,8,17,18]. Interestingly, even the ion channels are circadian-regulated, leading to a predicted day–night difference in cardiomyocyte electrophysiological properties [19]. Therefore, circadian rhythm variation may influence arrhythmic risk acting both on the substrate and on the trigger, which generates a different arrhythmic circadian pattern according to the primary underlying disease. As such, there are well-described circadian and seasonal variations in the distribution of arrhythmic events among acquired cardiovascular disorders [6,7,8], and the circadian and seasonal variations in the distribution of arrhythmic events in inherited cardiac disorders frequently differ from those observed in acquired cardiovascular disease [9,10,11,20,21,22]. Data on ARVC, however, are scarce.

ARVC is an inherited cardiac disorder that may result in arrhythmia and sudden cardiac death. It is primarily considered a disease of cell adhesion, as pathogenic mutations were detected in genes encoding proteins of the desmosome [23]. Several non-desmosomal genes were also implicated [23,24]. ARVC is characterised by long periods in which the disease remains quiescent (“concealed phase”), and then a life-threatening arrhythmia may suddenly occur. This phenomenon of “hot phases” is more difficult to explain and is probably due to unknown stimuli triggering acute inflammation. There are numerous clinical and experimental studies supporting the potential importance of myocardial inflammation as a determinant of life-threatening arrhythmia in patients with ARVC [25].

Alterations in calcium handling and in nervous system modulation may also represent the substrate for adrenergic-induced arrhythmia, particularly in the concealed phase of the disease [26,27,28]. Indeed, exercise was strongly associated with increased arrhythmic risk as well as disease progression [29].

Predicting risk, especially in the early phases, is a key and challenging component of the management of ARVC patients: the overlap with other inherited disorders, the well-established association of the disease progression and expression with exercise and the evidence of myocardial inflammation and of cardiac sympathetic dysfunction have highlighted the possible role of other genetic and non-genetic modifiers as well as of external factors in the arrhythmogenesis of the disease.

### 4.2. Circadian Distribution of the Arrhythmic Events in ARVC

**Figure 5:**
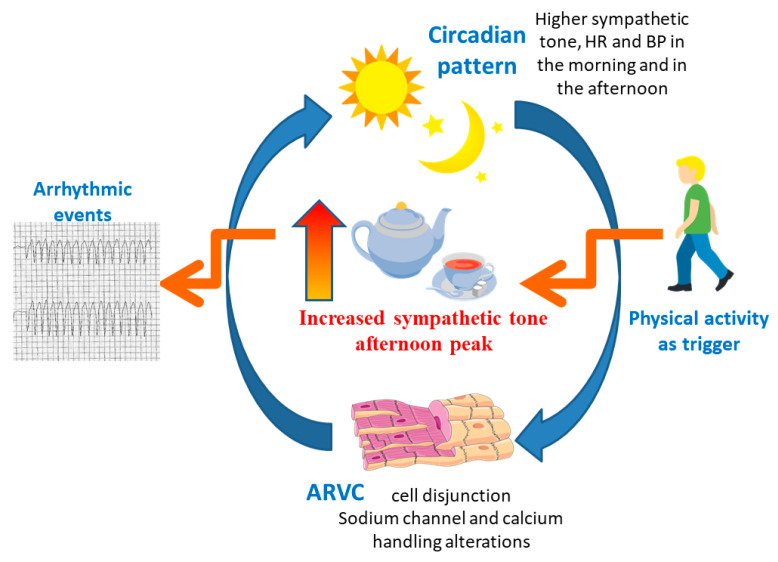
Illustration of the causes of the higher number of arrhythmic events in the afternoon. According to the circadian pattern, sympathetic tone (along with the heart rate and blood pressure) is higher in the early morning and in the late afternoon and lower during the evening and the night [32]. Desmosomal remodelling causes sodium channel and calcium handling alterations, with electromechanical alterations [26,31] that are part of the arrhythmic substrate. Ion channels and calcium handling are also circadian-regulated [19]. Their alteration causes a circadian rhythm malfunction with a further increase in sympathetic tone [28]. The threshold for arrhythmic events is, therefore, lower. Moreover, in the afternoon, the level of physical activity is higher than in the morning [33,34,35,36]. Therefore, increased physical activity may act as a trigger for ventricular arrhythmia in the afternoon. Free clip art from https://creazilla.com (accessed on 9 February 2022) and https://smart.servier.com (accessed on 9 February 2022).

Sudden cardiac deaths in the general population are mainly due to acute coronary disease, and, therefore, it is not surprising that the circadian variations in SCD and circadian variations in the arrhythmic events of acute cardiovascular and ischemic heart disease are similar, both of which are prevalent in the morning [7,8,30]. In contrast, in hypertrophic cardiomyopathy (HCM) and catecholaminergic polymorphic ventricular tachycardia (CPVT), arrhythmias occur more frequently in the afternoon [9,10,11,21], a very active phase of the day. Similarly, in our ARVC cohort, we observed a higher prevalence of arrhythmic events in the afternoon. This may reflect two aspects: (1) the structural genetic substrate leading to an alteration of the circadian rhythm with an increased sympathetic tone; (2) the pro-arrhythmic role of sympathetic tone and physical activity, especially in the early phase of the disease (Figure 5). In ARVC, desmosomal remodelling causes gap junction alteration and cell-to-cell disruption, affecting sodium channel function, which further influences calcium handling and mechanical coupling [26,31]. Cell disjunction, caused by the desmosomal remodelling and connexome subunits in areas of intense and constant mechanical stress, leads to myocyte death with an inflammatory response and fibro-fatty replacement, which causes heterogeneous conduction [26,31]. This heterogeneous conduction is able to favour re-entrant circuits and arrhythmia in the later stages of the disease, whilst, in the early stages, the electromechanical alterations represent the pro-arrhythmic substrate and are responsible for the altered response to mechanical stretch and adrenergic stimulation, even in the absence of significant fibro-fatty replacement [26,31]. Physical activity and exercise represent the perfect combination of mechanical stretch and adrenergic stimulation. Nevertheless, several studies have shown how, in ARVC patients, exercise acts both as a trigger for the arrhythmic events in the early stages and as an accelerator of the disease’s structural expression [29].

The normal circadian pattern of sympathetic tone, heart rate and blood pressure is bimodal, with two daytime peaks (one in the early morning and one in the late afternoon, around 6 pm) and a single nocturnal nadir [32]. However, the normal circadian pattern may be impaired in ARVC patients by desmosomal remodelling and sodium channel and calcium handling alterations, which are also circadian-regulated [17,18,19]. This malfunction may increase sympathetic activity, which is already higher in the afternoon, lowering the threshold for the arrhythmic events triggered by physical activity. Indeed, cardiac sympathetic dysfunction was demonstrated in genotype ARVC patients, and it was also associated with a higher incidence of ventricular arrhythmia recurrence [28]. In the afternoon, the level of physical activity is higher than in the morning [33,34,35,36]. The absence in our study of a higher incidence of arrhythmic events during the early hours of the morning, when there is the other heart rate and sympathetic tone peak, may be due to the reduced physical activity during those hours [33,34,35,36]. In ARVC, adrenergic stimulation and physical activity are known triggers for cardiac arrhythmia, and, therefore, it is not surprising that the peak of arrhythmia is in the afternoon, which is a very active phase of the day with a peak in sympathetic tone (Figure 5).

### 4.3. Seasonal Distribution of the Arrhythmic Events in ARVC Patients

Several studies report a higher frequency of sudden cardiac deaths, ventricular arrhythmia and cerebrocardiovascular ischemic events during the winter [5,6,37,38,39,40,41,42,43,44] and a similar pattern was also described in early repolarisation syndrome [45]. The reason for this pattern has been hypothesised to be multifactorial, involving both the hemodynamic response to cold temperature and environmental factors. Cold weather causes an increase in sympathetic tone, myocardial oxygen consumption, coagulation factors, red blood cell, platelet plasma count and the consequent increase in blood pressure and vasoconstriction [46]. Among the environmental factors, changes in habits, including diet and physical activity, and increased infections are the major factors. However, in a previous study on ARVC, a spring–summer pattern was highlighted [4]. This study took into account both living patients with appropriate ICD interventions and autopsied patients with SCD and found a seasonal peak in the summer months [4]. The reasons for this pattern was explained by meteorological factors, including increased variations in humidity and the high temperature, in the summer distribution of events in ARVC patients [4]. However, the study was conducted in Taiwan, where the meteorological factors differ from those in the Northern Hemisphere.

As such, in our study, we found a higher frequency of arrhythmic events during the winter and the lowest frequency in the summer. The reason for the winter pattern in ARVC patients, in our opinion, may be explained by the higher frequency of infections registered during the cold weather [47] and the role of inflammation in the disease expression [25].

Early post-mortem studies have shown that up to 70% of patients with ARVC have focal lymphocytic myocarditis [48,49,50]. As the presence of T-cell infiltrates in the absence of neutrophils rules out ischaemic injury, inflammation theory has been proposed: the fibro-fatty replacement may represent a healing process in the setting of chronic myocarditis with an immune-mediated injury and necrosis [48,51]. The detection of cardiotropic viruses in the myocardium of ARVC was claimed to support an infective aetiology of the disease [52,53,54,55,56], but the viral findings have not been confirmed [57]. However, in support of the inflammation role, there are numerous clinical reports of patients with myocarditis subsequently diagnosed with ARVC and of ARVC patients presenting with a myocarditis clinical picture, particularly during episodes of high disease activity [58,59,60,61,62]. The “hot phase” of the disease shares several clinical features with acute myocarditis, including symptoms, the release of myocardial enzymes, ECG changes and tissue-characterisation features. Recently, a study on 97 patients with acute myocarditis showed that a subgroup of them had pathogenic/likely pathogenic variants on the desmoplakin genes, and these were the patients with a more malignant phenotype [63]. Additionally, two independent studies have demonstrated the presence of autoantibodies to the heart and intercalated disk components in ARVC patients [64,65]: a positive correlation between disease severity, risk of arrhythmia and antibody titer was also noted [64]. Finally, in vitro [66,67] and in vivo [68,69,70] studies on ARVC animal models support the key role of inflammation in the disease expression. More recently, a human induced pluripotent stem cells study demonstrated immune activation, regardless of the presence of infiltrating inflammatory cells [71]. All this evidence has generated the hypothesis that a genetically vulnerable myocardium may predispose to myocarditis and that “hot phases” of the disease may be triggered by an inflammatory status and an abnormal inflammatory response. 

It is, therefore, not surprising that we found a higher frequency of arrhythmic events in ARVC patients during the winter time, when the possible inflammation triggers are more frequently observed.

### 4.4. Study Limitations

This is a retrospective study, and, as such, it carries the intrinsic limitation that detailed information on the circumstances of the events was not available for most of the patients; therefore, we cannot exclude that, when some arrhythmic events occurred, the patient was on holiday in a different country with a different temperature and/or time zone. Furthermore, we do not have data linking each arrhythmic event with a specific physical activity, work-shift, environmental temperature and humidity or with psychological stressors, nor do we know whether the patients were taking any stimulants (i.e., caffeine-based drinks). Finally, we do not have details about the ICD programming, so we cannot exclude that some arrhythmic events, mainly minor, were missed. However, all ICDs were transvenous ICDs, which reduces the variability in detection capabilities. 

## 5. Conclusions

One of the main challenging components in the management of ARVC patients is to predict the risk of arrhythmic events, especially in the early phases of the disease. We found a higher frequency of arrhythmic events in the afternoon and during the winter. This observation likely reflects the complex interplay between the circadian and seasonal cycles and environmental factors for the disease.

## Figures and Tables

**Table 1:** The 2010 Task Force Diagnostic criteria fulfilled by the 102 ARVC patients. * Numbers and categories (in parentheses) of fulfilled diagnostic criteria are reported. *a,* global or regional dysfunction and structural alterations; *b*, tissue characterisation of wall; *c*, repolarisation abnormalities; *d*, depolarisation abnormalities; *e*, arrhythmias; *f*, family history.

# Patient	Diagnostic Criteria *		# Patient	Diagnostic Criteria *	
	Major	Minor		Major	Minor
1	2 (*e*, *f*)	1 (*a*)	52	4 (*a, b, e, f*)	
2	2 (*e*, *f*)	1 (*c*)	53	2 (*a, e*)	
3	3 (*a*, *e, f*)	1 (*c*)	54	2 (*e, f*)	1 (*d*)
4	4 (*a, c, e*, *f*)	1 (*d*)	55	3 (*a, e, f*)	
5	2 (*e*, *f*)		56	1 (*c*)	2 (*e, f*)
6	3 (*c*, *e, f*)	1 (*a*)	57	3 (*a, e, f*)	2 (*c, d*)
7	2 (*c, f*)		58	2 (*a, c*)	1 (*d*)
8	3 (*a*, *c, f*)	1 (*e*)	59	2 (*e, f*)	
9	2 (*e*, *f*)		60	2 (*e, f*)	1 (*a*)
10	3 (*a*, *e, f*)		61	2 (*e, f*)	
11	2 (*a*, *f*)		62	2 (*e, f*)	
12	2 (*a*, *f*)		63	3 (*a, c, e*)	
13	2 (*e, f*)		64	2 (*e, f*)	1 (*c*)
14	2 (*a, c*)	2 (*e*, *d*)	65	3 (*a, c, e*)	
15	3 (*a*, *c, e*)	1 (*d*)	66	2 (*c, e*)	2 (*a, d*)
16	3 (*a*, *d, f*)	1 (*c*)	67	2 (d, f)	2 (*a, e*)
17	5 (*a*, *b, c, e, f*)	1 (*d*)	68	2 (*c, f*)	1 (*d*)
18	2 (*a, f*)	2 (*d, e*)	69	2 (*c, f*)	2 (*a, e*)
19	3 (*c*, *e, f*)		70	2 (*e, f*)	2 (*c, d*)
20	2 (*e, f*)		71	2 (*e, f*)	1 (*c*)
21	3 (*c*, *d, f*)	2 (*a, e*)	72	3 (*c, d, e*)	1 (*a*)
22	2 (*d*, *f*)	2 (*c, e*)	73	2 (*a, f*)	
23	3 (*a*, *d, f*)	1 (*e*)	74	3 (*a, e, f*)	
24	3 (*a, d, f*)	1 (*e*)	75	3 (*c, e, f*)	
25	2 (*e*, *f*)	1 (*c*)	76	2 (*a, f*)	1 (*d*)
26	2 (*d, f*)	3 (*a, c, e*)	77	4 (*a, c, e, f*)	
27	2 (*a, f*)	1 (*e*)	78	3 (*a, e, f*)	
28	2 (*e, f*)	1 (*a*)	79	3 (*a, e, f*)	1 (*c*)
29	2 (*e, f*)	1 (*d*)	80	3 (*a, c, f*)	
30	2 (*a, f*)	1 (*e*)	81	3 (*a, c, f*)	
31	2 (*a, d, f*)	1 (*e*)	82	2 (*a, e*)	
32	3 (*a, e, f*)	1 (*c*)	83	4 (*a, c, e, f*)	
33	3 (*a, e, f*)	1 (*c*)	84	2 (*c, e*)	1 (*a*)
34	1 (*f*)	3 (*c, d, e*)	85	3 (*a, c, e*)	
35	3 (*c, e, f*)		86	2 (*a, c*)	1 (*d*)
36	2 (*c, f*)		87	1 (*f*)	2 (*c, d*)
37	2 (*c, f*)		88	2 (*a, e*)	2 (*c, d*)
38	3 (*c, d, f*)	1 (*e*)	89	3 (*a, c, f*)	1 (*e*)
39	3 (*c, e, f*)	1 (*a*)	90	2 (*c, e*)	
40	1 (*f*)	3 (*a, d, e*)	91	2 (*e, f*)	
41	2 (e, *f*)	1 (*a*)	92	3 (*a, c, e*)	
42	2 (*c, e*)	1 (*d*)	93	2 (*a, e*)	1 (*c*)
43	2 (*c, f*)	2 (*a, e*)	94	4 (*b, c, e, f*)	1 (*a*)
44	5 (*a, c, d, e, f*)		95	2 (*e, f*)	1 (*c*)
45	2 (*a, f*)	1 (*d*)	96	2 (*a, e*)	
46	2 (*a, f*)	1 (*c*)	97	2 (*e, f*)	
47	2 (*e, f*)	2 (*c, d*)	98	3 (*a, c, f*)	2 (*d, e*)
48	3 (*a, e, f*)	1 (*c*)	99	4 (*a, c, e, f*)	
49	2 (*a, e*)	1 (*d*)	100	3 (*a, e, f*)	
50	3 (*a, e, f*)		101	2 (*a, f*)	1 (*e*)
51	1 (*f*)	2 (*e, d*)	102	3 (*a, c, f*)	2 (*d, e*)

**Table 2:** Demographic characteristics of the study population.

	Study Population 102	
Male/female	55 (54%)	47 (46%)
Caucasian/other	96(94%)	6 (6%)
Age at diagnosis/age at ICD implantation	40 ± 14	41 ± 15
Proband/family member	75 (74%)	27 (26%)

## Data Availability

The data presented in this study are available on request from the corresponding author. The data are not publicly available due to privacy reasons.
